# Management of Ankle Fracture Wounds Using Urinary Bladder Matrix: A Case Report

**DOI:** 10.7759/cureus.76584

**Published:** 2024-12-29

**Authors:** Mutaz Alenizi, Mohammed Tarabishi, Faisal Alayed, Omar Almutair

**Affiliations:** 1 Orthopedic Surgery, King Fahad Medical City, Riyadh, SAU; 2 College of Medicine, Qassim University, Buraydah, SAU

**Keywords:** acellular scaffold, ankle fractures, ankle trauma, postoperative wounds, wound infections

## Abstract

Ankle fractures, often accompanied by other injuries and complications, pose a significant healthcare burden due to their high incidence and associated treatment challenges. This case report investigates the use of Cytal™ Wound Matrix, derived from urinary bladder matrix (UBM), in managing postoperative complications following open reduction and internal fixation (ORIF) of a trimalleolar ankle fracture.

A 57-year-old male with a history of hypertension sustained a trimalleolar ankle fracture following a road traffic accident. Surgical intervention (ORIF) was performed after swelling subsided. Three weeks post-surgery, the patient developed wound-edge necrosis and superficial infection, identified as methicillin-sensitive *Staphylococcus aureus* (MSSA). The wound was debrided, and Cytal™ Wound Matrix was applied. The patient received intravenous cefazolin, followed by oral Augmentin after two weeks.

The application of UBM over the wound showed significant improvement, with tissue remodeling observed within two weeks. The infection was successfully controlled with targeted antibiotics, and no additional plastic surgery interventions were required.

This case highlights the efficacy of UBM in promoting wound healing, controlling infection, and preventing further invasive procedures, suggesting its potential as a valuable adjunct in managing complex orthopedic wounds. Further research with larger cohorts is necessary to fully evaluate UBM's clinical effectiveness and cost-benefit profile.

## Introduction

An ankle fracture refers to the disruption of one or more of the three osseous structures constituting the ankle joint: the tibia, fibula, and talus. These fractures are frequently accompanied by additional injuries, including open wounds, ligamentous ruptures, and, in some cases, neurovascular damage. Ankle fractures are among the most prevalent fractures in adults, with an incidence rate of up to 174 cases per 100,000 individuals annually [1].

A 2022 study conducted in the United States reported that approximately 23.4% of all ankle fractures necessitated surgical intervention [2]. The high incidence of ankle fractures, coupled with the associated morbidity, treatment costs, and the significant loss of workdays for affected individuals, underscores the substantial societal burden posed by these injuries.

Certain ankle fractures generally require surgical intervention, with open reduction and internal fixation (ORIF) being the most commonly performed procedure. Like all surgeries, patients may experience postoperative complications, with superficial wound infections and wound-edge necrosis being among the most prevalent [3]. Those with such complications often require extensive wound care, prolonged courses of oral antibiotics, and in some cases, advanced plastic surgery procedures. However, recent advancements in medical technologies, such as the Cytal™ Wound Matrix, have shown promise as a therapeutic agent for promoting healing in various types of both surgical and non-surgical unhealed wounds.

Cytal™ Wound Matrix is an acellular matrix derived from the natural urinary bladder matrix (UBM), containing bioactive antimicrobial peptides produced through the proteolysis of collagens and elastin present in the extracellular matrix (ECM) [4]. This matrix supports the remodeling of functional, site-appropriate tissue and preserves the integrity of the epithelial basement membrane, thereby promoting cellular infiltration and capillary ingrowth.

The UBM matrix scaffold is a sterile, lyophilized product that has been thoroughly cleaned of urothelial cells and adipose tissue, virtually eliminating the risk of immunogenic rejection. This makes it particularly suitable for large-scale applications and for use in immunocompromised patients. Before applying Cytal™ Wound Matrix, the wound bed should be properly prepared and debrided to remove any necrotic tissue. During the treatment period, the wound must be maintained in a moist environment and covered with a non-adherent primary dressing.

In this paper, we have assessed and investigated the effectiveness of using UBM in the management of ankle fracture postoperative wound-edge necrosis, dehiscence, and superficial surgical site infections.

## Case presentation

The patient is a 57-year-old male with a history of hypertension, for which he is currently prescribed amlodipine 5 mg daily. He was involved in a road traffic accident in November 2023. Following stabilization according to the Advanced Trauma Life Support protocol, clinical examination and imaging revealed a closed right ankle trimalleolar fracture. The patient presented with significant right ankle swelling, but no open wounds, and his neurovascular examination was unremarkable. Surgical intervention was planned once the swelling subsided, allowing for optimal wound closure and minimizing the risk of potential complications.

**Figure 1:**
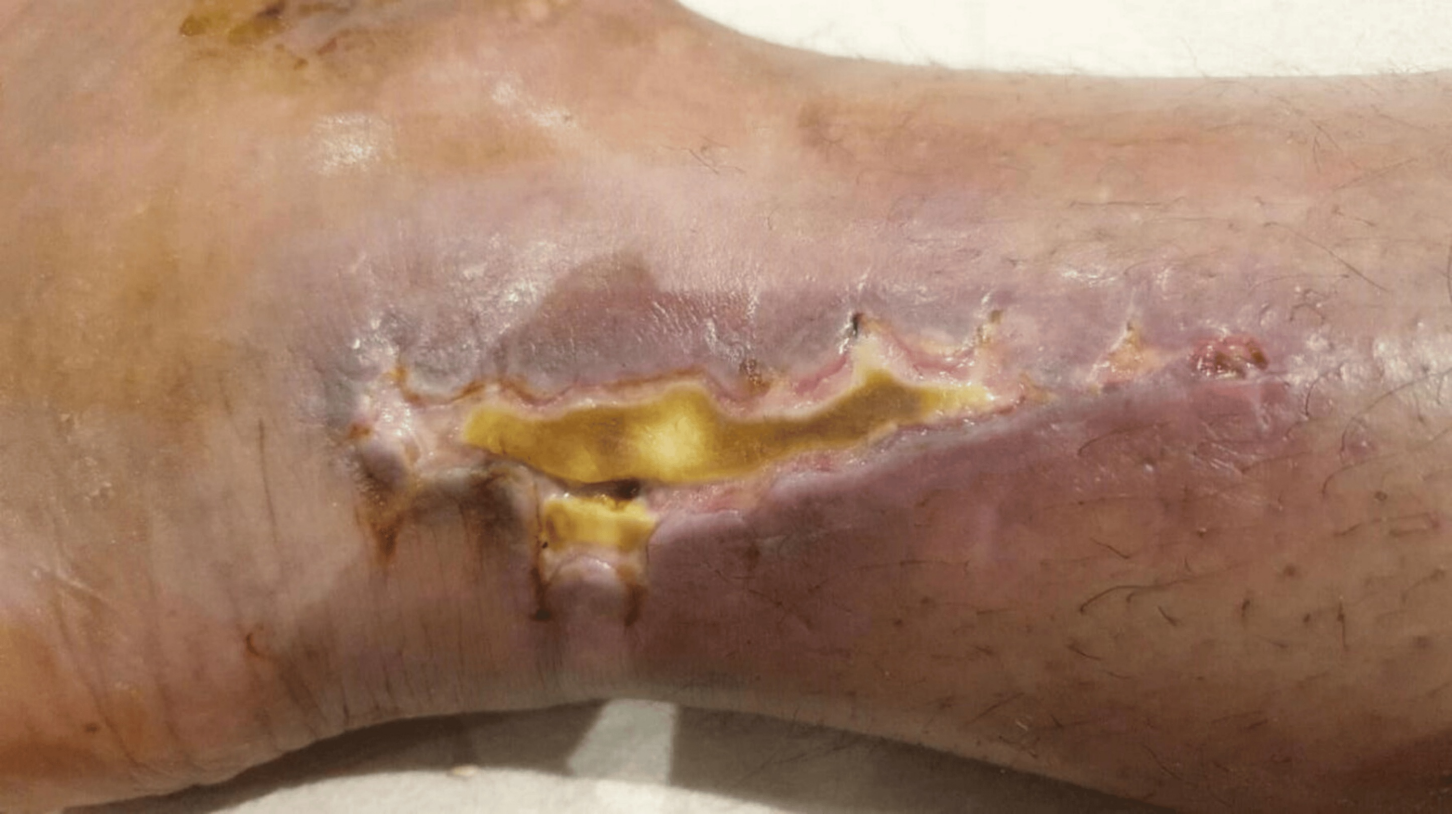
The patient’s right ankle wound before applying Cytal Wound Matrix. The patient’s right ankle wound before applying Cytal Wound Matrix, three weeks after open reduction and internal fixation of right ankle trimalleolar fracture, showing postoperative surgical site infection, pus discharges, wound edge necrosis, and slough tissues.

On 17/12/2023, the patient underwent right ankle ORIF. The procedure involved posteromedial, anteromedial, and anterolateral approaches, with a 5 cm interval between every two incisions. The surgery was uneventful with no intraoperative complications. However, three weeks post-surgery, the patient’s wound began to show signs of complications, with pus discharge emerging from the wound. Additionally, approximately a 3 cm area of wound-edge necrosis was noted over the posteromedial incision site (Figure 1).

**Figure 2:**
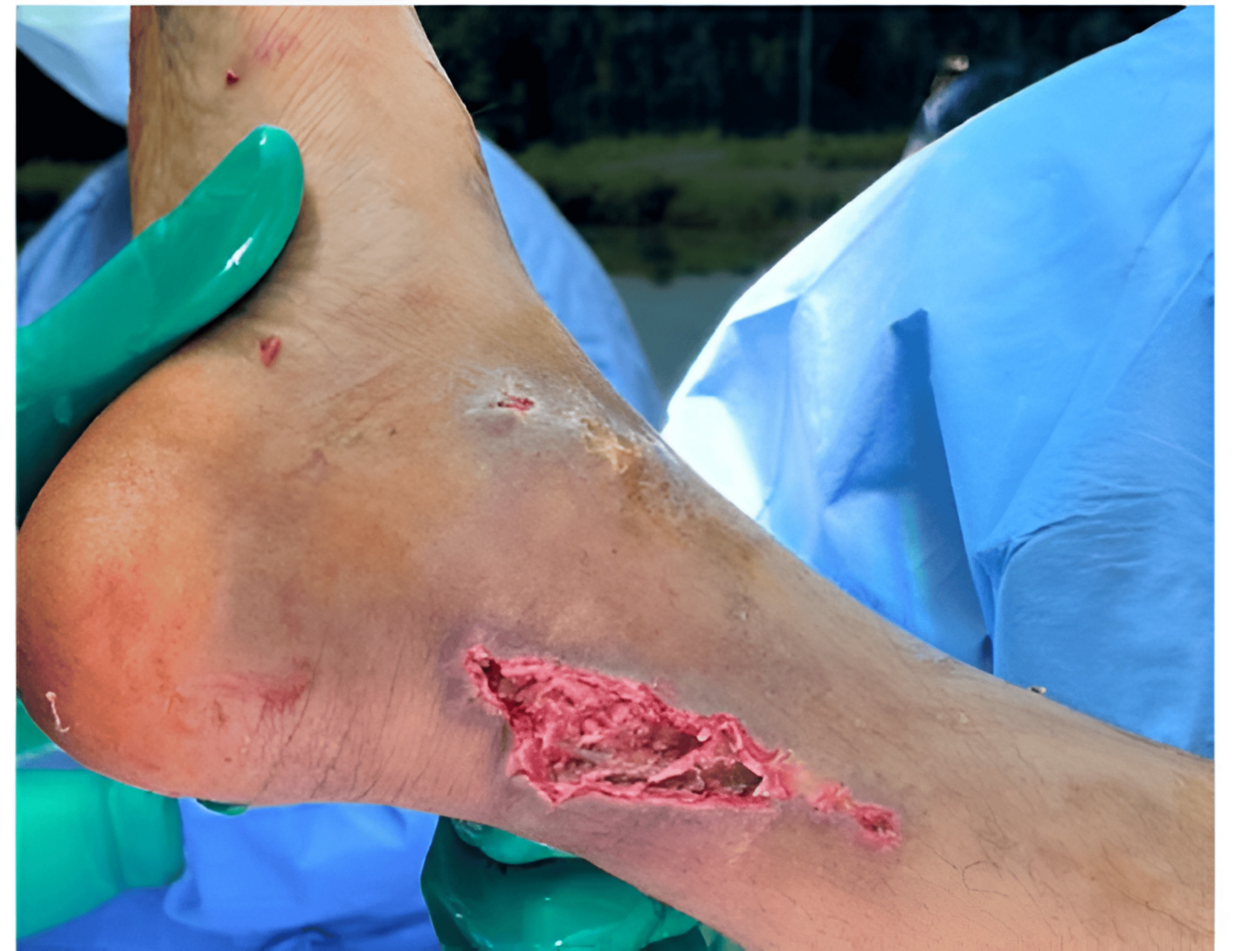
The patient's right ankle wound after irrigation and debridement. The patient's right ankle wound after irrigation and debridement showing 4 x 3 cm skin and subcutaneous tissue loss that could not be primarily sutured.

The patient underwent irrigation and debridement on February 4th, 2024. Throughout the procedure, the wound was thoroughly cleansed using povidone iodine, followed by irrigation with 6 liters of normal saline to ensure optimal cleansing. All necrotic tissue was carefully debrided, and tissue samples were collected and sent for culture and sensitivity analysis (Figure 2).

**Figure 3:**
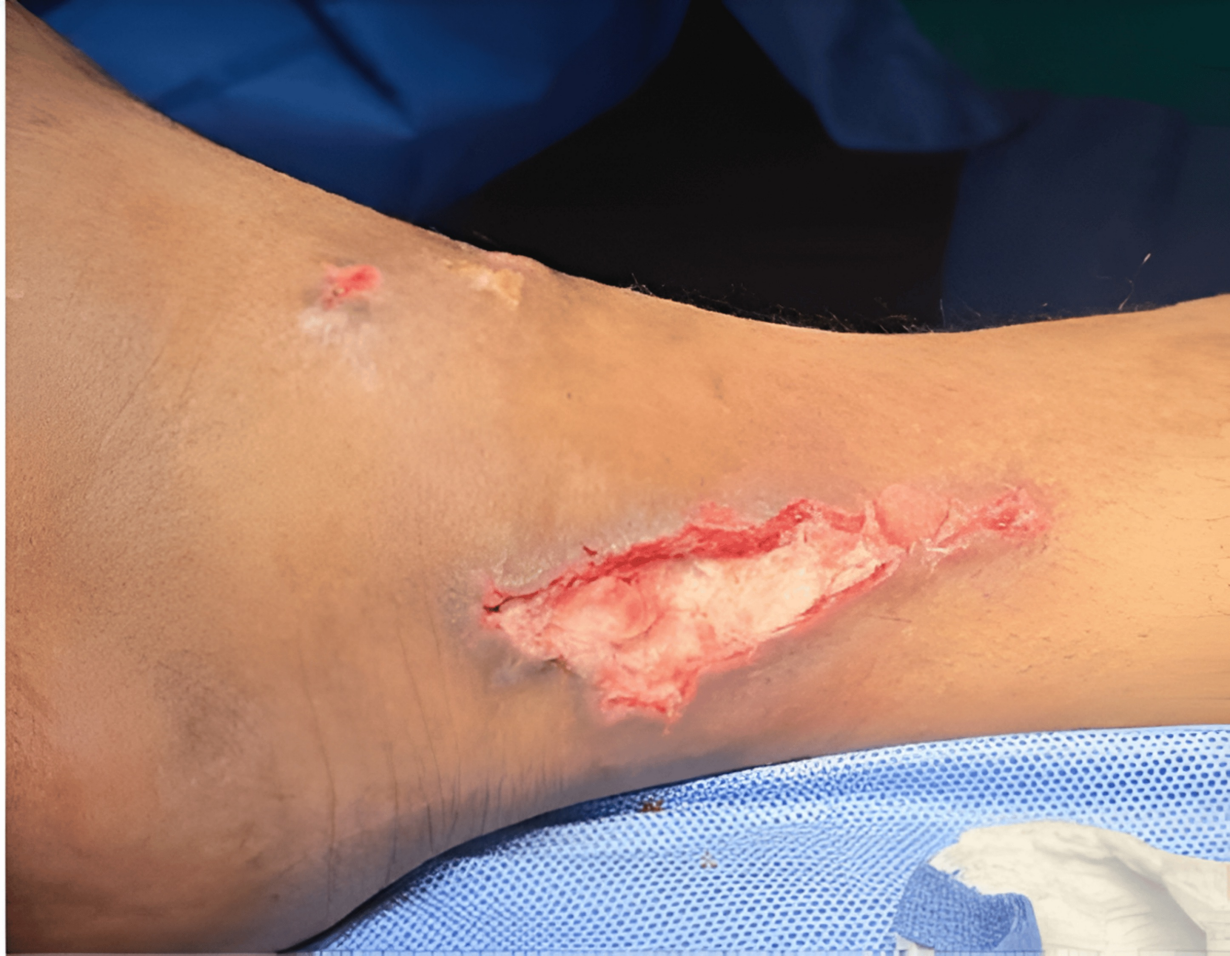
The patient's right ankle wound after applying Cytal Wound Matrix. The patient's right ankle wound after applying Cytal Wound Matrix, showing how the Cytal Wound Matrix sheets were fitted nicely inside the wound and filling all defective areas.

Additionally, Cytal™ Wound Matrix was applied to the wound (Figure 3).

Four days later, the culture results confirmed methicillin-sensitive *Staphylococcus aureus* (MSSA). Following a review by the infectious disease team, intravenous cefazolin (1 gram every 8 hours) was initiated. The dressing was changed weekly, with the removal of the old matrix and the application of a new one at each dressing change.

**Figure 4:**
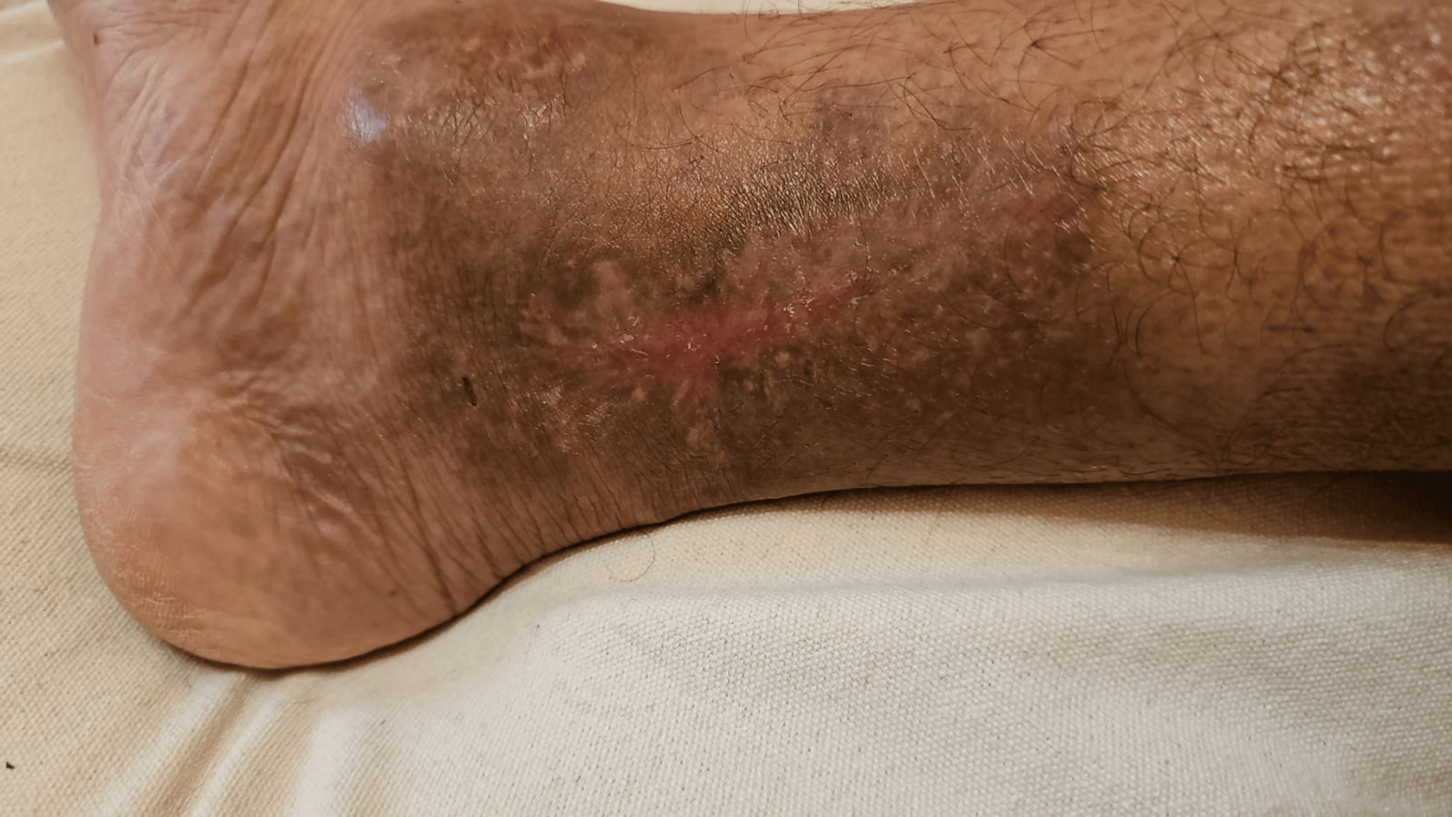
The patient's right ankle wound three months after Cytal Wound Matrix application. The patient's right ankle wound after three months since applying Cytal Wound Matrix showing complete wound healing, with no signs of infection.

After two weeks, significant improvement was observed, including wound margin shrinkage and evidence of tissue remodeling. After observing improvement in the patient’s condition, the infectious disease team transitioned treatment to oral Augmentin 1 gram twice daily for two weeks. The patient was subsequently discharged with instructions for weekly follow-ups in our clinic. Three months later, the outcomes were remarkable, and fortunately, there was no need to involve the plastic surgery team for additional procedures to address the wound defect (Figure 4).

## Discussion

This case highlights the effective use of Cytal™ Wound Matrix (a UBM) in managing postoperative wound complications, including wound-edge necrosis and superficial surgical site infections, following ORIF of a trimalleolar ankle fracture. The patient's remarkable recovery without the need for additional plastic surgery interventions underscores the potential benefits of this advanced wound care modality.

The management of wound complications following orthopedic surgeries remains a significant clinical challenge. In this case, traditional wound management techniques, such as irrigation, debridement, and culture-guided antibiotic therapy, were complemented by the application of the Cytal™ Wound Matrix. The observed outcomes evidenced by wound margin shrinkage, tissue remodeling, and the prevention of long-term complications align with the findings from prior studies demonstrating the efficacy of UBM in promoting wound healing. For example, studies on UBM have highlighted its role in accelerating tissue repair by supporting cellular infiltration, capillary ingrowth, and functional tissue remodeling [4]. These properties were evident in the rapid wound improvement observed in this patient within two weeks of treatment initiation.

The selection of Cytal™ Wound Matrix in this case was driven by its unique characteristics, including its bioactive antimicrobial peptides and preserved ECM integrity. These features make UBM particularly valuable in cases complicated by infection, as demonstrated by the resolution of an MSSA infection. The use of UBM in conjunction with targeted antibiotic therapy likely created a synergistic effect, facilitating both infection control and tissue regeneration. Importantly, the ability to avoid more invasive interventions, such as flap-based plastic surgery, reduced the patient’s recovery time, hospital stay, and overall healthcare costs, thereby mitigating the societal burden often associated with complex ankle fracture management.

Despite the promising outcomes, certain limitations must be acknowledged. This report details a single case, which limits the generalizability of the findings. Additionally, the patient's baseline health status and the absence of significant comorbidities apart from hypertension may have contributed to the favorable outcome. Patients with compromised vascular supply or other comorbidities may respond differently to this treatment approach.

Future research should focus on larger, controlled studies to better evaluate the efficacy and cost-effectiveness of UBM in various wound types and patient populations. Comparative studies between UBM and other advanced wound care products could further elucidate its relative benefits. Additionally, investigations into the long-term durability of tissue remodeled using UBM would be invaluable in understanding its full potential in orthopedic and reconstructive surgery.

To summarize, our case demonstrated the utility of Cytal™ Wound Matrix in managing postoperative wound complications in ankle fractures. Its ability to promote healing, minimize infection, and reduce the need for additional surgical interventions highlights its potential as a valuable adjunct in wound care. With further research, this technology may become a standard component of advanced wound management protocols.

## Conclusions

In conclusion, this case report demonstrates the successful application of Cytal™ Wound Matrix (UBM) in the management of postoperative wound complications following a trimalleolar ankle fracture. The use of UBM facilitated significant wound healing, tissue remodeling, and infection control, preventing the need for further invasive procedures. The patient showed remarkable recovery, with no additional interventions required from the plastic surgery team.

Given the promising outcomes observed, UBM appears to be a valuable adjunct in the management of complex surgical wounds, particularly in patients at risk for wound-edge necrosis or infection. While further research involving larger cohorts and diverse patient populations is needed to confirm its widespread efficacy, this case highlights the potential of UBM to improve patient outcomes, reduce healthcare costs, and minimize the need for extensive surgical interventions. In light of these findings, UBM could represent a significant advancement in the field of wound care for orthopedic and other high-risk surgeries.
